# Diphtheria

**Published:** 1873-01

**Authors:** W. T. Taylor

**Affiliations:** Fishersville, Kentucky


### DIPHTHERIA.

BY W. T. TAYLOR, M.D., FISHERSVILLE, KENTUCKY.

Twelve or fifteen years ago, when diphtheria first made its appearance in this State, in an epidemic form, little was known as to its nature, treatment or pathology. It was believed that the disease was primarily local, and the routine practice consisted, among other things, in the application of strong caustic solutions to the throat, with the double view of their local action on the diseased membrane, and the removal of false membranes from the passages.

The section in which I first saw the disease was preëminently miasmatic, being a low, flat country, extending back from the Ohio river; and malarial fevers were prevailing during that year to an extent even greater than usual. The epidemic was terribly fatal, due doubtless, in a great degree, to the miasmatic location in which it prevailed. Indeed, the peculiar impress of this mysterious agent could be recognized, if not as a cause, certainly as a controlling agent, in a vast majority of the cases.

We do not propose, in this paper, to discuss the intimate nature or true pathology of diphtheria, but whatever these may be, one thing is patent to every observer, *that certain habits and constitutions are especially obnoxious to the disease,* and these are principally from among those which go to make up the long list of cachexies, scrofulous, tubercular, etc. On the part of such persons, there seems to be an inherent predisposition to the disease, which renders them peculiarly liable to an attack from almost any cause capable of considerably disturbing the vital functions.

In the epidemic of which I write, as has already been intimated, the almost universal practice was caustic solutions to the throat, and it is to this practice we wish specially to call attention. I, with the others, kept this up for a time, not knowing anything better to do, and feeling that we ought to do something. But after awhile, it began to be observed that, in almost every case, an aggravation of all the throat symptoms followed the application of the caustic—an increased swelling of the cervical, sub-maxillary and neighboring glands, attended by a corresponding extension of the disease within and below the fauces, by declension of power and increased difficulty of breathing and swallowing.

The true nature of the disease gradually dawned upon us, that the throat affection derived "all its importance from its being a local manifestation of a serious constitutional disease," and that our practice was based on a false hypothesis, and that, instead of benefitting our patients, we were absolutely doing them a mischief.

As an example of the treatment then adopted, and the result, I transcribe *one* case from my note-book, which may be taken as a fair sample of this class of cases. I do this the more willingly, as I fear this sort of practice is still resorted to in many localities, and I cannot forbear to express my unqualified condemnation of this indiscriminate use of caustics, as being no less false in theory than cruel in practice, subjecting, as it does, the sufferer to an ordeal as painful as it is useless. Either the whole theory of the constitutional nature of diphtheria is false, or the practice of cauterizing "gangrenous throats" is without excuse.

Ofie P——, a child of ten years, of good constitution, and not subject to any of the influences supposed to be favorable to the development of the disease—having the advantage of the very best of nursing—her father a physician of good attainments and much experience.

I was called to see her the third day after she was attacked; found her apparently in not much pain, breathing comfortably without noise; swallowing freely and without difficulty anything that was given to her; soft, cool skin, small pulse beating 110. The cervical and sub-maxillary glands were much swollen. She could open her mouth very well, and, by means of a spoon, a very good view of the throat was obtained. The soft palate was projecting, strongly convex on to the base of the tongue; the swelling terminating on the hard palate, within half an inch of the front teeth; it was covered in patches with the characteristic false membrane, and exuded copiously a jelly-like, tenacious fibrin. By drawing forward and pressing down the tongue, the margin of the false palate could be seen, with its thick, swollen edges dipping down in the pharynx; the uvula pale-red and free from disease, or at most slightly œdematous. The tonsils were much swollen, and matted with effusion of false membrane and fibrin.

I was told that the disease was decidedly progressive, the swelling being greater than it was six hours before.

She had had quinine in 2 grs. doses every four or five hours, with potass. chlor. solution *ad. lib.*

It was resolved to apply a strong solution of argent. nit. (18 grs. to ℥ i.), which was done after removing as much of the exudation as possible.

Quinine and chlorate potass. continued with Port wine. In four or five hours, the breathing became noisy from the blocking up of the posterior nares, from increased swelling at the back part of the velum, together with effusion of fibrinous secretion, spotted with false membrane, causing a corresponding difficulty of ingress of air by the mouth. Inhalation of vinegar gave much relief.

*Fourth Day.*—Noisy breathing considerably diminished. Patient slept three or four hours during the night. She had taken her medicine, food and wine, but with more difficulty than yesterday, and once the fluid returned by the nostrils. The false membrane and fibrin secreted in less quantity, but the cervical and sublingual glands much more swollen. General symptoms about as yesterday. It was felt that the caustic solution had done good in one direction, and mischief in another. The solution was weakened to grs. x. to ℥ i. of water, and applied.

*Fifth Day.*—No sleep; increased difficulty in swallowing; considerable accumulation behind the fauces; occasional smothered cough; cervical and neighboring glands immensely swollen; the mouth could not be opened sufficiently to examine throat; laryngeal spasm had occurred once, and was overcome by inhalation. At the request of the family, I remained. In two hours, another spasm, overcome by same means. Sonorous respiration followed, and in an hour a third spasm of the glottis closed the scene.

In the above case, I tried hard to believe that the application of the caustic had no connection with the rapid aggravation of the symptoms, and did partially persuade myself that the local treatment was the best possible under the circumstances; but subsequent experience has convinced me that any treatment which causes rapidly-increasing swelling of the glands is bad, and sure to be attended by corresponding extension of the disease within and below the fauces. Even if the local treatment did no mischief, it could result in no permanent good. And the reason is obvions enough. "The great red current itself is poisoned at the fount," and the plague-spot in the throat is only a local evidence of disease in the veins, in the arteries and in the heart, at every feeble contraction of which incipient death is pumped, not only into the throat, but throughout the entire system.

As a result of my experience of the disease, in this and other localities, I long ago became convinced that diphtheria is of an erysipelatous nature, and requires mainly the same general plan of treatment as erysipelas.

The result of this treatment, too, would go far to corroborate this view, as in the epidemic to which reference has been made the mortality was reduced more than fifty per cent. after the treatment was adopted. In the years that have passed since then, I have not had occasion either to change my theory or practice; on the contrary, as the disease has apparently domiciled itself among us, and we expect its autumnal appearance now with as much certainty as we do the "chills," and as opportunities for observation and examination have multiplied, my belief has grown stronger and stronger until it has culminated into absolute certainty.

Whether I can give to others satisfactory reasons for the "faith' that is within me," I will not now stop to inquire, but will hazard the hope that, at some future time, I may be permitted to at least make the attempt. For the present, I will merely give a synopsis of the general plan of treatment.

To begin, a mild purgative is given, sufficient to thoroughly evacuate the bowels; and in this connection I will say, that I do not share the view so many have taken, as to the inadmissibility of mercurials in diphtheria. By this I do not mean that I would give them in all cases, but I do mean that I have found many cases in which mercury, in stimulating the hepatic secretion and emptying the bowels, has proved essentially beneficial. When these are accomplished, however, I know of no further indications for its use.

After the bowels are evacuated, by which time, usually, the initial fever begins to decline, and the characteristic depression becomes manifest, the following may be given:

℞.—Tinct. ferri. mur., ℥ ss—℥ i; quinia sul., grs. x—xx; syr. simplex, ℥ ij; M. and give one teaspoonful in substance, or diluted with water, every two, three or four hours. If it is thought better, the iron and quinine may be given separately, every four hours, alternately. Brandy, whisky or wine when indicated; beef tea, animal broth, and abundance of fresh air.

The only local application is a moderately strong solution of the tinct. ferri. mur., used as a gargle, and repeated several times in the twenty-four hours. External applications I regard as of but little benefit—always bearing in mind that the throat disease is but a local evidence of blood poison, and our only hope of curing the patient is based upon the success we have of eliminating the *materies morbi* through the emunctory organs, and sustain the strength until such time as the vital forces shall resume their normal power.

When there is much difficulty of breathing, or tendency to spasm of the *larynx* or *glottis*, inhalation from a hot infusion of chamomile flowers, to which, if there be much fetor, may be added a few drops of carbolic acid, or of liq. calcis chlor. ( ʒ ij to ℥ xv.)

Of course, no definite plan of treatment can be given for each individual case, as complications will continually meet us at almost every step, requiring a modification of the above. The good sense and skill and watchfulness of the physician must ever be on the alert, to meet the indications as they arise. The only point I insist upon, after the bowels are cleared out, is, that the tinct. ferri. mur. be made the *base* of treatment.

From what we have said, we assume the following:

1. That diphtheria, in its prominent characteristics, has much in common with erysipelas, and that the treatment should be addressed to the elimination of the *materies morbi.*

2. That the tinct. ferri. mur., in an eminent degree, meets the indications, both in its local and constitutional effects.

3. That the use of powerful caustics is based on false premises, and cannot be too strongly condemned.

---

IODINE may be used as an air-disinfectant in a very simple manner—first suggested by Dr. B. W. Richardson. Solid iodine is merely exposed in glass or porcelain vessels in different parts of the room. The iodine vapor is given off at ordinary temperatures. This is a very efficient mode of attaining a constant disinfection.

